# COVID-19-Related Changes to Pregnant People's Work-Plans Increase Prenatal Depression

**DOI:** 10.3389/fgwh.2021.639429

**Published:** 2021-04-21

**Authors:** Margaret Sherin, Theresa E. Gildner, Zaneta M. Thayer

**Affiliations:** ^1^Geisel School of Medicine, Dartmouth College, Hanover, NH, United States; ^2^Department of Anthropology, Dartmouth College, Hanover, NH, United States; ^3^Department of Anthropology, Washington University in St. Louis, St. Louis, MO, United States

**Keywords:** Coronavirus, pregnant workers, perinatal depression screening, paid family leave, Family and Medical Leave Act

## Abstract

The COVID-19 pandemic has caused unprecedented rates of unemployment in the United States. Pregnant workers may be especially affected as they are over-represented in low-wage service and hospitality industries impacted by the pandemic. We surveyed an online convenience sample of currently working pregnant people living in the U.S. (*n* = 1,417) to determine whether COVID-19-related changes to how long individuals planned to work during their pregnancy, and uncertainty about these changes, were associated with prenatal depression. As hypothesized, both COVID-19-related work-plan changes (OR = 1.81, 95% CI 1.36–2.42, *p* < 0.001) and uncertainty about the precise nature of these changes (OR = 2.62, 95% CI 1.14–6.0, *p* = 0.022) were associated with significantly higher odds of a clinically-significant depression score. These effects appeared to be even greater among individuals who continued working outside the home during the pandemic. Since the U.S. is one of the few countries in the world that does not guarantee paid parental leave, pregnant people may be forced to choose between keeping their jobs and risking infection during the COVID-19 pandemic. Our results demonstrate a need for immediate suspension of the eligibility requirements for the Family and Medical Leave Act and/or universal access to both paid family leave and prenatal depression screening. This would help to alleviate these concerns and provide pregnant people with more options while preserving their employment status and financial security.

## Introduction

The COVID-19 pandemic has drastically affected employment in the U.S., with national unemployment rates hovering around 6.7% as of December 2020 after reaching a high of 14.7% in April 2020 (1, 2). Women, especially those that are pregnant, are over-represented in low-wage industries impacted by the pandemic (e.g., service and hospitality), leaving them vulnerable to changes in their work-plans during their pregnancy (3–5). Moreover, the three most common jobs held by pregnant women (elementary school teachers, nurses, and home health aides) put them at significant risk for infection (3). In the absence of universal paid parental leave and strict eligibility requirements for the federal 1993 Family and Medical Leave Act (FMLA), pregnant persons working outside the home may be forced to choose between keeping their job and risking infection (6–10), leading them to feel like they have little control over their circumstances.

Perceived lack of control over life events can substantially impact mental health, increasing depression risk (11–14). For example, pregnant individuals reporting a low sense of control over triggers such as stressful life events and lower income have been found to have a higher likelihood of perinatal depression (15). It is therefore reasonable to expect that unplanned alterations to work-plans and uncertainty about the nature of these changes due to the COVID-19 pandemic may elevate depression risk among pregnant persons. However, this has yet to be fully explored, despite the fact that pregnant people display elevated depression risk. Depression during and after pregnancy is more common than in the general population, affecting one in seven women in high-income countries (15). This may be due to higher rates of depression in women than men in general, as well as increased life demands and hormonal changes during pregnancy and the postpartum period.

Even still, perinatal depression is often underreported due to stigma (15, 16) and a failure of health care providers to screen pregnant people for depression (17). The CDC reports that about one in five pregnant people are not asked about depression symptoms during a prenatal visit (17). Recent evidence suggests that perinatal depression symptomatology has become even more common during the COVID-19 pandemic (18–20). These changes appear to be linked with factors including disruptions to daily exercise routines, social isolation, financial stress, and fears of COVID-19's impact on mothers and their infants' long-term health, highlighting the need for increased perinatal depression screening and treatment (18–22). Given the high percentage of individuals with prenatal depression who go undiagnosed and untreated, there is a need to improve depression screening and identify risk factors, especially during the COVID-19 pandemic.

Considering this background, the present study assesses whether changes and uncertainty surrounding work-plans during the COVID-19 pandemic significantly predict the likelihood of prenatal depression among pregnant persons living in the U.S. Specifically, we examined whether pandemic-related changes to how long individuals intended to work during their pregnancy were associated with prenatal depression. We also examined whether uncertainty about changes to work-plans was associated with prenatal depression. We hypothesized that currently working individuals reporting changes to how long they planned to work in pregnancy or uncertainty about their work-plans during the COVID-19 pandemic would have significantly higher depression scores. Information on the effects of the COVID-19 pandemic on pregnant people's work-plans and associated depression risk can help guide both the development of comprehensive national policies on paid parental leave, and universal screening and referral protocols for perinatal depression.

## Materials and Methods

Data come from the COVID-19 and Reproductive Effects (CARE) study, an online survey that was administered to a convenience sample recruited primarily through social media (i.e., Twitter and Facebook) and via dissemination to U.S.-based contacts working in maternal health. Surveys were completed between April 16th−30th 2020. The target population for the CARE study was pregnant people aged 18 years or older living in the United States, while the present analysis focuses specifically on those participants who were working at the time of survey completion. This study received ethical approval from Dartmouth College (STUDY00032045). Informed consent was collected by participants clicking a box saying that they consent to the information provided on the consent form. The survey was administered in REDCap, which automatically captures survey responses.

Completion of the survey was voluntary, and participants were allowed to skip any questions they did not want to answer. Only individuals who completed the survey (went through to the end of the questionnaire, even if they were missing data on individual questions) were included in the analysis. Of 2,467 people who consented to take the survey, 1,970 completed it (80%). Of the complete surveys, 1,600 participants were currently working and therefore eligible for inclusion in these analyses.

### COVID-19 Pandemic Effects on Work-Plans

Participants were asked “Has the pandemic changed your plans for how long you plan to work during your pregnancy?” (yes/no).

### Work-Plan Uncertainty

If participants reported that COVID-19 had changed their work-plans, they were prompted to qualitatively describe how their work-plans had been affected. A subset of 348 participants provided a qualitative response describing these changes. Given that work-plan uncertainty has been linked with increased emotional distress (23), these 348 qualitative responses were assessed to identify participants who were uncertain about the precise nature of changes to work-plans (e.g., whether or not they would stop working earlier than planned due to fear of getting COVID-19 or an inability to continue working entirely from home).

### Work Location

Participants who reported that they were currently working were asked to identify their work location (from home; outside the home; or both).

### Depression Symptoms

Depression symptoms were screened for using the Edinburgh Postnatal Depression Survey (EPDS) (24). The EPDS is considered to be the gold standard perinatal depression measure and is the most widely used validated screening tool worldwide [e.g., (25, 26)]. Depression symptoms were analyzed according to clinically significant prenatal depression criteria for pregnant persons (cut point ≥ 15) (27).

### Age

Participants self-reported their age in years.

### Education

Participants selected their highest completed education from the following options: Some high school, no diploma (1) High school graduate, diploma or the equivalent (for example: GED) (2) Some college credit, no degree (3) Trade/technical/vocational training (4) Associate degree (5) Bachelor's degree (6) Master's degree (7) Professional degree (8) Doctorate degree (9). A composite education variable was created for analysis: less than a bachelor's degree, a bachelor's degree, or a degree beyond a bachelor's degree.

### Household Income

Participants indicated their annual household income (USD) from the following options: < $10,000 (1); $10,000–19,999 (2); $20,000–34,999 (3); $35,000–49,999 (4); $50,000–74,999 (5); $75,000–99,999 (6); $100,000+ (7). A composite household income variable was created for analysis: < $49,999, $50,000–99,999, and $100,000+.

### Race/Ethnicity

Race/ethnicity were self-reported and measured according to the Office of Management and Budget Standards (28). Native Hawaiian/Pacific Islander participants were re-classified as “Other” due to a small sample size (*N* = 3).

### Current Gestational Week

Participants indicated their current gestational week.

### High-Risk Pregnancy

Participants were categorized as high risk if they reported that they had been classified as “high-risk” by their maternity care provider or if they were aged 35 or older.

### Self-Reported Health

Participants were asked whether they would describe their health as poor, fair, good, or excellent. This was re-categorized into good/excellent vs. poor/fair.

### Statistical Analysis

Data analyses were conducted using Stata 15.1. All continuous variables exhibited normal distributions, with skewness values within ~±0.5 and kurtosis values within ~±3. Multicollinearity was not detected between any variables; all VIF values were in an acceptable range of 1.03–1.75. Sample descriptive statistics were calculated and bivariate analyses were conducted to evaluate significant differences in study covariates according to COVID-19-associated work-plan changes.

A multivariate logistic regression was used to evaluate whether work-plan changes predicted a clinically significant depression score (EPDS ≥ 15; yes/no). The model was adjusted for maternal age, education, income, week of pregnancy at time of survey, self-rated health, race/ethnicity, working outside the home, and “high-risk” pregnancy. After analyzing this relationship in the entire sample, we then stratified the analysis according to whether participants were working entirely from home during the pandemic or working outside the home. We then repeated this analysis process (i.e., multivariate logistic regression including the same covariates within the complete sample, and then stratified by work location) to evaluate whether there was an association between depression and work-plan uncertainty.

## Results

In total, 1,417 participants were eligible for the study (i.e., reported they were currently working) and were not missing data for study variables and were therefore included in the analysis. Study participants were a mean of 31.7 years old (SD = 4.2) and 25.8 weeks pregnant (SD = 8.8) at the time of survey completion. The study sample was 87.0% White (*N* = 1223), 5.6% Hispanic/Latina (*N* = 79), 1.6% African American (*N* = 23), 3.5% Asian (*N* = 49), 0.6% American Indian/Alaskan Native (*N* = 8), and 1.8% Other (*N* = 25). Over one-third (34.7%, *N* = 491) of the study population had a college education, and nearly one-half (49.4%, *N* = 700) of the study population had a degree beyond a college education. When asked about household income, 8.2% of respondents (*N* = 116) reported earning < $49,999 annually, 34.7% (*N* = 491) reported earning between $50–99,000, and 61.6% (*N* = 873) reported earning $100,000+ (Table 1). Moreover, 26.2% of study participants (*N* = 372) reported that they experienced a COVID-19-related work-plan change (Table 1). Additionally, among the 348 participants who described how their work-plans had been altered by COVID-19, 30 individuals explicitly stated that they were uncertain how the pandemic would alter their work-plans.

**Table 1:** Descriptive statistics.

	**Total sample (*N* = 1,417)**	**COVID-19 related change in work-plans (*N* = 372)**	**No COVID-19 related change in work-plans (*N* = 1,045)**	*p*-value^*^
**Age**	31.7 (4.2)	31.3 (4.1)	31.8 (4.2)	0.10
**Weeks pregnant**	25.8 (8.8)	27.6 (8.2)	25.2 (9.0)	** <0.001**
**Race/ethnicity**				0.27
White	1,233 (87.0%)	322 (86.6%)	911 (87.2%)	
Hispanic/Latino	79 (5.6%)	25 (6.7%)	54 (5.2%)	
African American	23 (1.6%)	2 (0.5%)	21 (2.0%)	
Asian	49 (3.5%)	15 (4.0%)	34 (3.3%)	
American Indian/Alaska Native	8 (0.6%)	3 (0.8%)	5 (0.5%)	
Other	25 (1.8%)	5 (1.3%)	20 (1.9%)	
**Household Income**				** <0.001**
<49,999	116 (8.2%)	48 (12.9%)	68 (6.5%)	
$50–99,000	428 (30.2%)	119 (32.0%)	309 (29.6%)	
$100,000+	873 (61.6%)	205 (55.1%)	668 (63.9%)	
**Education**				**0.008**
Less than a college education	226 (16.0%)	78 (21.0%)	148 (14.2%)	
College education	491 (34.7%)	124 (33.3%)	367 (35.1%)	
Degree beyond College education	700 (49.4%)	170 (45.7%)	530 (50.7%)	
**Self-rated health**				0.44
Poor/Fair	91 (6.4%)	27 (7.3%)	64 (6.1%)	
Good/Excellent	1,326 (93.6%)	345 (92.7%)	981 (93.9%)	
**High-risk pregnancy**	498 (35.1%)	126 (33.9%)	372 (35.6%)	0.55
**Previous birth**	608 (42.9%)	160 (43.0%)	448 (42.9%)	0.96
**Provider type**				0.78
Obstetrician	1,148 (81.0%)	306 (82.3%)	842 (80.6%)	
Midwife	244 (17.2%)	60 (16.1%)	184 (17.6%)	
Other	25 (1.8%)	6 (1.6%)	19 (1.8%)	
**Current work location**				** <0.001**
Home	1,039 (73.3%)	226 (60.8%)	813 (77.8%)	
Outside home	286 (20.2%)	123 (33.0%)	163 (15.6%)	
Both in the home/outside the home	92 (6.5%)	23 (6.2%)	69 (6.6%)	
**Depression symptoms (EPDS, 0–30)**	10.5 (5.2)	12.0 (5.1)	9.9 (5.1)	** <0.001**

*Values with p < 0.05 are shown in bold*.

* *P-value represents significant differences in each variable according to whether or not COVID-19 affected people's work plans. T-tests were used for continuous variables, chi-squared tests for categorical variables*.

*Mean (SD) reported for continuous variables, N (%) for categorical variables*.

In bivariate analyses, participants who experienced a COVID-19-related work-plan change reported lower household incomes [$\chi_{\left(2\right)}^{2}$ = 17.70, *p* < 0.001], were less educated [$\chi_{\left(2\right)}^{2}$ = 9.62, *p* = 0.008], were farther along in pregnancy [t_(1415)_ = −4.57, *p* < 0.001), and were more likely to continue to work outside the home during COVID-19 [$\chi_{\left(2\right)}^{2}$ = 52.41, *p* < 0.001] compared to individuals who did not experience a COVID-19-related work-plan change. There were no statistically significant differences in maternal age [t_(1415)_ = 1.66, *p* = 0.10], race/ethnicity $\chi_{\left(5\right)}^{2}$ = 6.37, *p* = 0.27], high-risk pregnancy [$\chi_{\left(1\right)}^{2}$ = 0.36, *p* = 0.55], previous birth [$\chi_{\left(1\right)}^{2}$ = 0.002, *p* = 0.96], self-rated health [$\chi_{\left(1\right)}^{2}$ = 0.59, *p* = 0.44] or provider type [$\chi_{\left(2\right)}^{2}$ = 0.51, *p* = 0.76] between individuals reporting a work-plan change and those reporting no change (Table 1).

**Figure 1:**
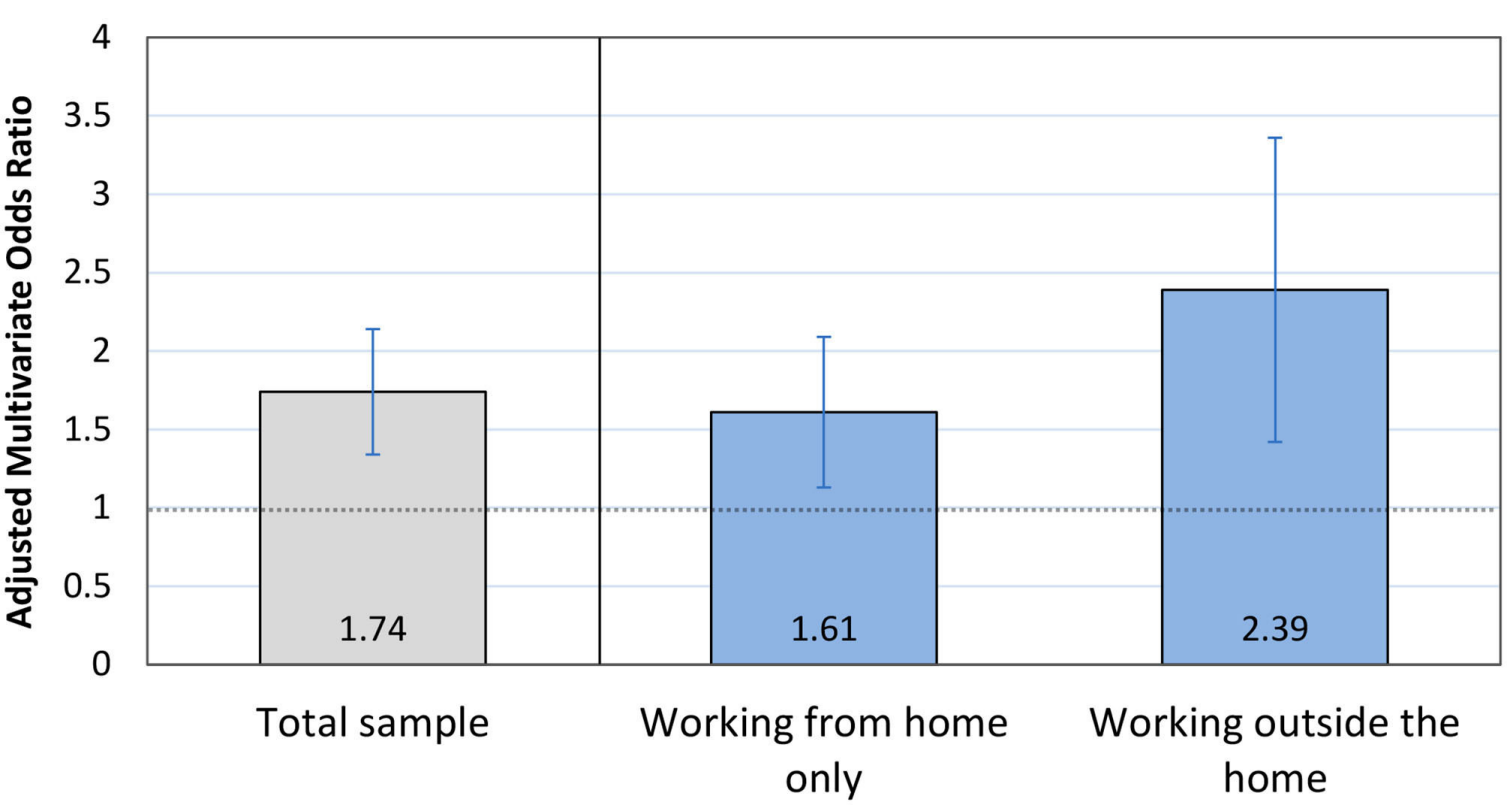
Prenatal depression and COVID-19-related work-plan changes. Odds ratio and 95% confidence interval from multivariate regression model predicting clinically significant depression symptoms in response to experiencing a work-plan change. Results are shown within the total sample (in gray); and then stratified according to whether individuals worked entirely from home or outside of the home during the pandemic (in blue).

Participants who experienced a COVID-19-related work-plan change had a significantly higher mean EPDS score (12.0) compared to individuals who did not report a COVID-19-related work-plan change [EPDS = 9.9; t_(1415)_ = −6.81, *p* < 0.001]. In multivariate logistic regression models using an EPDS score ≥ 15 as the clinical cutoff for depression and adjusting for covariates, participants reporting a work-plan change were significantly more likely to exhibit a depression score above the clinical cutoff point compared to those reporting no work-plan change (OR = 1.81, 95% CI 1.36–2.42, *p* < 0.001, Table 2). When stratifying the sample according to whether participants worked entirely from home or outside the home, work-plan change was significantly associated with an increased likelihood of depression in both models, but the OR appeared larger for those working outside the home (working outside the home: OR = 2.39, 95% CI = 1.42–4.05, *p* = 0.001; working from home only: OR = 1.61, 95% CI 1.13–2.29, *p* = 0.007, Figure 1).

**Table 2:** Regression results for association between work-plan changes and clinically significant depression (Model 1) and uncertainty about work-plan changes and depression (Model 2).

	**Model 1 (*N* = 1,417)**	**Model 2 (*N* = 348)**
	**OR (95% CI)**	**OR (95% CI)**
Work-plan change	1.81 (1.36–2.42)^*^	–
Uncertainty about work-plan change	–	2.62 (1.14–6.00)^*^
**Provider type**		
Obstetrician	REF	REF
Midwife	0.98 (0.69–1.39)	0.56 (0.27–1.17)
Other	0.77 (0.26–2.29)	1.32 (0.23–7.72)
**Race/ethnicity**		
White	REF	REF
Hispanic/Latino	1.43 (0.86–2.40)	0.94 (0.35–2.48)
African American	1.14 (0.40–3.20)	Omitted
Asian	0.27 (0.09–0.79)^*^	0.59 (0.15–2.31)
American Indian/Alaska Native	1.30 (0.26–6.62)	1.64 (0.13–18.78)
Other	1.66 (0.67–4.10)	2.18 (0.33–14.24)
**Income**		
<49,999	REF	REF
$50–99,000	0.78 (0.48–1.27)	0.89 (0.39–2.02)
$100,000+	0.72 (0.44–1.17)	1.04 (0.45–2.38)
**Education**		
Less than a college education	REF	REF
College education	0.82 (0.55–1.21)	0.64 (0.32–1.26)
Degree beyond college education	0.69 (0.46–1.03)	0.59 (0.30–1.16)
No previous birth	0.93 (0.71–1.22)	0.90 (0.53–1.53)
High risk pregnancy	1.05 (0.76–1.46)	1.08 (0.59–7.95)
Weeks pregnant	1.00 (0.98–1.01)	0.99 (0.96–1.02)
Maternal age	0.98 (0.94–1.02)	0.97 (0.90–1.05)
**Current health**		
Poor/Fair	REF	REF
Good/Excellent	0.29 (0.19–0.47)^*^	0.50 (0.22–1.18)
Adjusted model R2	0.05	0.04

*Values with p < 0.05 are shown in bold*.

* *p < 0.05*.

**Figure 2:**
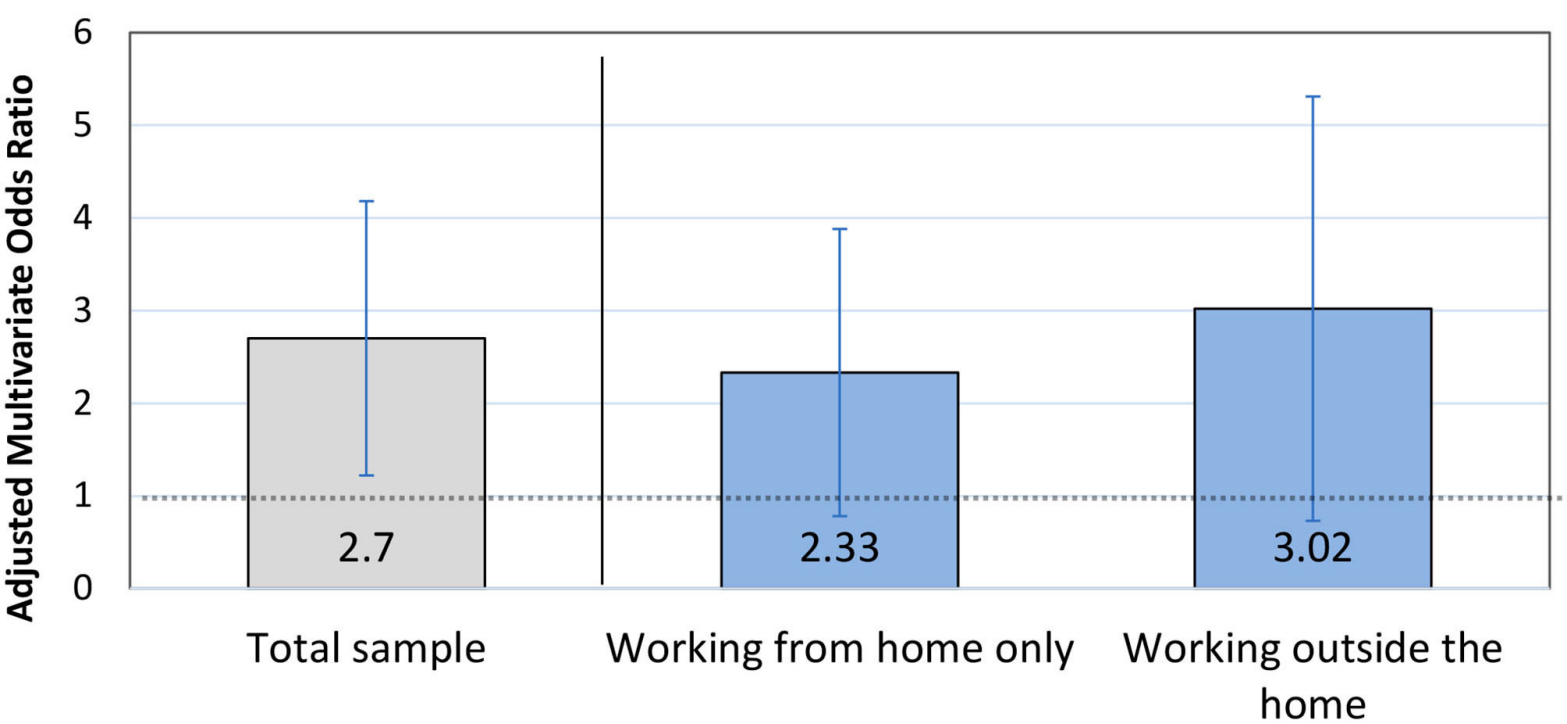
Prenatal depression and uncertainty surrounding COVID-19-related work-plan changes. Odds ratio and 95% confidence interval from multivariate regression model predicting clinically significant depression symptoms among participants in response to being unsure about how the pandemic would affect their work-plans. Results are shown within the total sample (in gray); and then stratified according to whether individuals worked entirely from home or outside of the home during the pandemic (in blue).

Likewise, uncertainty about the nature of the work-plan change was significantly associated with an increased likelihood of having a depression score above the clinical cutoff point (OR = 2.62, 95% CI 1.15–6.0, *p* = 0.022, Table 2). When stratifying the sample according to whether participants worked entirely from home or outside the home, the coefficients were similar to or greater than that for the total sample but were no long statistically significant (working outside the home: OR = 3.02, 95% CI = 0.73–12.6, *p* = 0.13; working entirely from home: OR = 2.33, 95% CI = 0.78–6.92, *p* = 0.13) (Figure 2). This non-significant finding may be due to reduced statistical power resulting from only a subset of the sample reporting on the exact nature of work-plan changes. Self-rated health was the only covariate that was significantly associated with depression in adjusted models; respondents who reported good/excellent health had a lower likelihood of depression regardless of work-plan changes (Model 1: OR = 0.29, 95% CI 0.19–0.47, *p* < 0.001) (Table 2).

## Discussion

The aim of our study was to examine whether COVID-19-related changes to how long pregnant people intended to work during their pregnancy were associated with prenatal depression. Pregnant people who reported that COVID-19 had affected how long they planned to work during pregnancy were significantly more likely to exhibit signs of clinical depression, as were individuals who were unsure how their work-plans would be affected by COVID-19. These effects appeared to be particularly strong among individuals who continued to work outside of the home during the pandemic. Cumulatively, our findings support the study hypotheses and are consistent with earlier work. Previous research conducted among working pregnant people living in the U.S. indicates that maintaining a sense of control bolsters mental health during the transition to parenthood (29). Keeton et al. also found that this protective sense of control includes perceived ability to manage work schedules (29). Likewise, reports of “serious difficulties at work” have been linked with prenatal depression risk (30). These findings and the results of the present study suggest that work disruptions may elevate maternal depression risk.

Pregnant workers are especially vulnerable to COVID-19-related work disruptions. The three most common occupations for pregnant women are elementary school teachers, nurses, and home health aides, all of which have been considered essential during the COVID-19 pandemic and may put individuals at significant risk of contracting disease (3). Additionally, more than one in five pregnant workers are employed in low-wage jobs, which often have inflexible scheduling (impairing ability to attend doctor appointments) and generally lack paid sick leave or work-from-home options (3–5). These factors render pregnant workers in essential and low-wage positions more susceptible to contracting COVID-19 and thus increase their likelihood of having a work-plan change during the pandemic, either because they become sick or fear becoming sick.

Additionally, uncertainty surrounding future work-plans appears to be an important determinant of depression risk among pregnant people, which is consistent with previous findings that perceived lack of control over life events is central to the development of depression (12, 14, 15, 31, 32). Therefore, in response to rising perinatal depression rates during the pandemic, obstetric care providers can use the results from this analysis to better identify depression risk factors during COVID-19 and use this information to better screen patients. For instance, the results of our analysis suggest that clinicians should consider adding screening questions related to work-plan disruptions (either resulting from COVID-19 or other factors), as they could signify a risk factor for depression. Moreover, clinicians and medical researchers should consider how uncertainty related to a range of factors—including work-plans, financial situations, and even pregnancy outcomes—may impact maternal mental health. Documenting common sources of uncertainty may help healthcare providers and researchers design more effective interventions to address these underlying issues (e.g., offer targeted information and resources relevant to a particular source of uncertainty, thereby helping the affected individual regain some sense of control).

Unfortunately, even though mental health concerns are increasing during the pandemic, in-person clinical screens for depression have decreased as most prenatal appointments are done remotely by telehealth (4, 18–20, 33). The American College of Obstetricians and Gynecologists recommends that obstetric care providers screen patients for depression and anxiety at least once during the perinatal period using a standardized, validated tool (15). Many studies (4, 34, 35) have argued for obstetricians to go beyond this recommendation and make depression screening a routine part of prenatal care, as screening is critical to avoid adverse outcomes for mother and baby and to reduce postpartum depression risk, a leading cause of maternal mortality (16, 34–37).

One way to mediate the effects of COVID-19-related work-plan changes and prenatal depression would be through universal paid family leave, which could alleviate anxiety caused by choosing to work while risking infection (4). The U.S. is one of the few countries in the world that does not guarantee paid parental leave, despite the benefits associated with leave (e.g., reduced cesarean section rates and lower infant mortality) (6, 9). The FMLA gives workers 12 weeks of *unpaid* time off, but only ~60% of workers are actually eligible (7, 9, 10). Employer provision of any *paid* family leave (PFL) is voluntary and more common among high-paying occupations; in 2018, only 16% of employees had access to PFL (7, 8, 10). Policy recommendations could therefore include instituting a universal paid family leave policy and/or temporarily suspending FMLA eligibility requirements (5). Future research can also investigate whether states and countries with more favorable parental leave policies have had better mental health outcomes during the COVID-19 pandemic relative to those without such policies.

Despite the strengths of this study, including the large sample size and wide distribution of participants across the U.S., our study was limited by the nature of self-reported data (38). Future work should test these associations using more objective measures designed to explicitly capture types of work-plan changes and the uncertainty surrounding these changes [e.g., providing a list of possible work-plan changes and asking participants to select the response(s) that accurately described their situation]. Additionally, as the survey was distributed through social media, it did not involve random sampling, despite attempts to distribute the survey to a diverse set of maternal-health organizations in various states. Our study population was less diverse than the U.S. birthing population, with study participants more likely to be non-Hispanic White and to report higher education and income levels than national averages (39). Another limitation was that prenatal participant data were not available on social support received or relevant mental health history (e.g., current psychotropic treatment, previous depression diagnoses, or family history of mental illness), variables that have been linked with prenatal depression risk e.g., (30, 40). Finally, individuals with severe depression may have been less likely to complete the study survey.

Our analysis also only included currently working individuals, excluding anyone who stopped working before our study began. We focused on currently working participants to evaluate the relationship between work-plan changes and prenatal depression, since depression risk among individuals who had already stopped working could be impacted by changes in routine, financial stress (21), and other factors not directly related to anticipated work-plan changes. Additionally, while respondents qualitatively described working more or less during pregnancy due to the pandemic, this information was not systematically collected so was not included in the present analysis.

More research is needed to assess the effects of work-plan changes and uncertainty during the COVID-19 pandemic in more diverse study populations, especially as Black and Latinx individuals are more likely to hold low-wage occupations affected by the pandemic (3). Moreover, populations who do not speak English or have reliable internet access may be particularly affected by the absence of in-person perinatal depression screens due to language and technology barriers (4). Longitudinal research is also needed to establish directionality in the relationship between COVID-19-related work-plan changes and depression.

Our study found that COVID-19-related work-plan changes and work-plan uncertainty were associated with depression, independent of risk factors including income and education. These results suggest a need for increased mental health screening during the pandemic by providers. Additionally, increased access to FMLA and universal PFL may help reduce stress both during and after the pandemic.

## Data Availability Statement

Study data are available from the corresponding author upon reasonable request. Requests can be made through the CARE study website (https://sites.dartmouth.edu/care2020/).

## Ethics Statement

This study received ethical approval from Dartmouth College (STUDY00032045). Study information (e.g., study summary, purpose, benefits, risks, and privacy protection details) was provided to all participants prior their completion of the survey. Informed consent was collected by asking potential participants to click a box saying that they had read the study information and consented to participating in the study.

## Author Contributions

MS: conceptualized and wrote the manuscript. TG and ZT: study design, revised, and wrote the manuscript. All authors contributed to the article and approved the submitted version.

## Conflict of Interest

The authors declare that the research was conducted in the absence of any commercial or financial relationships that could be construed as a potential conflict of interest.
